# Observation of grating diffraction radiation at the KEK LUCX facility

**DOI:** 10.1038/s41598-020-63462-1

**Published:** 2020-05-05

**Authors:** A. Aryshev, A. P. Potylitsyn, G. A. Naumenko, M. Shevelev, D. Shkitov, L. G. Sukhikh, N. Terunuma, J. Urakawa

**Affiliations:** 1 KEK: High Energy Accelerator Research Organization, 1-1 Oho, Ibaraki, 305-0801 Tsukuba, Japan; 2 Tomsk Polytechnic University, Lenin ave. 30, Tomsk, 634050 Russian Federation Russia

**Keywords:** Experimental particle physics, Experimental particle physics, Terahertz optics, Terahertz optics

## Abstract

The development of linac–based narrow–band THz sources with sub–picosecond, $\mu J$-level radiation pulses is in demand from the scientific community. Intrinsically monochromatic emitters such as coherent Smith–Purcell radiation sources appear as natural candidates. However, the lack of broad spectral tunability continues to stimulate active research in this field. We hereby present the first experimental investigation of coherent grating diffraction radiation (GDR), for which comparable radiation intensity with central frequency fine–tuning in a much wider spectral range has been confirmed. Additionally, the approach allows for bandwidth selection at the same central frequency. The experimental validation of performance included the basic spectral, spatial and polarization properties. The discussion of the comparison between GDR intensity and other coherent radiation sources is also presented. These results further strengthen the foundation for the design of a tabletop wide–range tunable quasi–monochromatic or multi–colour radiation source in the GHz–THz frequency range.

## Introduction

Various applications of THz radiation demand a high–brilliance monochromatic source with tunable characteristics such as spectral range, pulse duration, polarization, and directivity^1–4^. Semi–conventional linac–based THz sources can provide sub-picosecond radiation pulses at approximately the hundred $nJ$-level with a continuous spectrum up to $1$ THz^5–9^. However, in many applied investigations, narrow–band sources at this energy level are desired^10–14^. Evidently, to achieve this objective, the usage of a monochromator is required. However, approaches offering more output energy are related to the utilization of sources based on radiation mechanisms which are intrinsically monochromatic; for instance, coherent Smith-Purcell radiation (SPR)^15^ or coherent Cherenkov radiation from dielectric lined waveguides^16^. Typically, spectral line fine-tuning of SPR-based sources is performed by output photon angular selection, i.e., by detection aperture positioning^17^ or by the rotation of an additional mirror upon parallel passage of an electron beam near a grating^18^. Superior spectral tuning can only be accomplished by the replacement of the grating with another having a different profile or period^19^. In the refs. ^20,21^ initial proposals to use non–parallel orientation of a grating relative to a beam to change the frequency of the SPR spectral lines for a given outgoing photon angle were presented. In this case, the position of the collimator or aperture, which provided the radiation beam spectral acceptance, was fixed. Moreover, in ref. ^20^ the generalized dispersion relation for the inclined grating was obtained as:

1 $$\lambda =\frac{d}{k}(\frac{cos\,\eta }{\beta }-cos\,(\theta -\eta )),$$

where $\lambda$ is the radiation wavelength, $d$ is the grating period, $k$ is the diffraction order, $\beta$ is the electron velocity in light units, $\eta$ is the grating orientation angle, and $\theta$ is the observation angle. For a charge trajectory parallel to the grating ($\eta =0$) Eq. 1 coincides with the well-known SPR dispersion relation $\lambda =\frac{d}{k}({\beta }^{-1}-{\rm{c}}{\rm{o}}{\rm{s}}\,\theta )$. It is important to note that Eq. 1 is also valid for larger grating orientation angles that are typically unreachable for SPR generation experiments due to the longitudinal grating dimensions and the small distance to the electron beam. However, implementation of large angle radiation emission may be considered in a “diffraction radiation”–like arrangement. This should lead to different radiation polarization maps, intensities and directivity profiles, although the approach offers wide–range monochromatic spectral tunability which still follows Eq. 1. Radiation generated in this case, by analogy with the grating transition radiation^22–24^, also has significant spectral tunability but without the drawback of the destructive interaction of the electron beam with the grating material. This radiation is referred to as the “grating diffraction radiation” (GDR).

In this report, we demonstrate the first experimental observation of coherent GDR which includes the investigation of the basic spectral, spatial and polarization properties in addition to a discussion on GDR intensity in comparison with coherent SPR and coherent diffraction radiation (CDR). The result improves the prospects of designing a tabletop wide–range tunable quasi–monochromatic or multi–colour radiation source in the GHz–THz frequency range.

## Results

**Figure 1:**
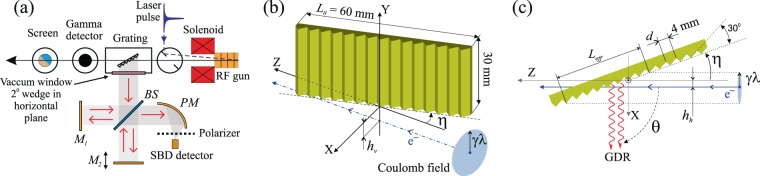
Schematics of the experimental layout (**a**) and grating geometry (**b,c**). Abbreviations: ${M}_{1}$ - fixed interferometer mirror, ${M}_{2}$ - movable mirror, $BS$ - beam splitter, $PM$ - parabolic mirror with a focal length of $152$ mm, ${L}_{0}$ - grating length, ${L}_{eff}$ - interaction length, ${h}_{v}$ - vertical impact-parameter, ${h}_{h}$ - horizontal impact-parameter, $\eta$ - grating inclination angle, $\theta$ - observation angle, $d$ - grating period, ☉ - grating rotation axis in XZ plane.

Details of the experimental geometry are illustrated in Fig. 1. The Coulomb field of the relativistic charge with the effective radius $\gamma \lambda$ ($\gamma$ is the Lorentz-factor and $\lambda$ is the radiation wavelength) interacts with a tilted periodic structure on the length ${L}_{eff}=2\gamma \lambda /sin\,\eta$ for $\eta  > {\eta }_{c}=arcsin\mathrm{[2}\gamma \lambda /{L}_{0}]$ and on the length ${L}_{eff}={L}_{0}$ if $\eta \le {\eta }_{c}$, assuming the axis of rotation in the center of the grating and ${h}_{h} < \gamma \lambda$, where ${L}_{0}$ is the grating length and ${h}_{h}$ is the horizontal impact-parameter. If the relation ${N}_{eff}={L}_{eff}/d\gg 1$ is satisfied, one can expect that the resulting radiation would become quasi-monochromatic and the full width at half maximum (FWHM) bandwidth can be estimated as^25^:

2 $$\Delta \lambda /\lambda =\mathrm{0.89/}k{N}_{eff}=0.89d\,\sin \,\eta \mathrm{/2}k\gamma \lambda \mathrm{}.$$

The monochromaticity estimation from the above–given equation is valid only for a small capture angle of the detection system $\Delta \theta \ll 0.89\lambda /{L}_{eff}\,\sin (\theta -\eta )$, and if the chosen grating parameters and geometry satisfy the condition ${N}_{0}\ge {N}_{eff}\gg 1$, where ${N}_{0}$ is the number of grating periods. The factor $0.89$ comes from the Fourier transform of the periodical function (grating periods), shaped by the rectangular window representing the finite grating length^26^.

**Figure 2:**
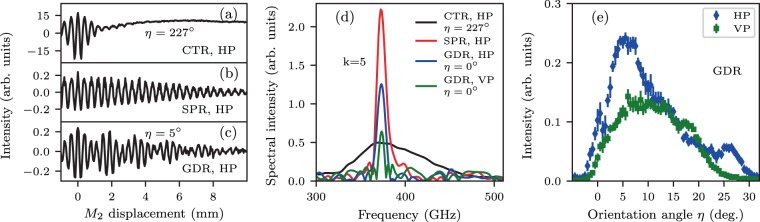
Zoom-in of the measured auto–correlation curves of CTR (**a**), SPR (**b**) $k=5$, ${h}_{v}=-\,15$ mm, ${h}_{h}=0.5$ mm, and GDR horizontal polarization (**c**) for $\eta ={5}^{\circ }$, $k=5$. (***d***) Comparison of the CTR spectrum and SPR, GDR spectra taken for ${h}_{v}=0.5$ mm and ${h}_{h}=0.5$ mm. (**e**) GDR horizontal and vertical polarization angular distributions taken for ${h}_{v}=0.5$ mm.

In the present investigation, we measured the spectral–angular distribution and polarization properties of the radiation produced by the $8$ MeV, $25$ pC single electron bunch with $0.15$ mm rms length passing below the $4$ mm period, $30\times 60$ mm^2^, ${N}_{0}=15$, echelette profile grating (Fig. 1b,c). To reveal the actual full bandwidth of the spectrometer system $\Delta {\nu }_{exp}=140\pm 1$ GHz, the broadband coherent transition radiation (CTR) spectrum from the flat surface on the rear of the grating plate was measured. The CDR was generated from the same surface, but when the target’s vertical position was set to allow for the electron beam passage below the target. It is important to mention that two impact-parameters, the horizontal ${h}_{h}$ and the vertical ${h}_{v}$ *(h*_*v*_ < 0 for the CTR and *h*_*v*_ > 0 for the CDR), are considered as explained in the Methods section. Figure 2 shows a comparison of the typical charge–normalized auto–correlation dependencies (*a-c*), measured spectra (*d*) taken for the same observation angle $\theta ={90}^{\circ }$ and angular acceptance of the detection system $\Delta \theta ={1.6}^{\circ }$, and GDR orientation dependencies (*e*). As can be seen in Fig. 2d, the Schottky Barrier Diode (SBD) detector has a full bandwidth of $320-460$ GHz^25,27^ which limits the overall spectral sensitivity of the system. For a direct spectral intensity comparison, the spectrum of the coherent SPR, horizontal polarization was measured when the electron beam passed near the grating in SPR geometry: ${h}_{h}=0.5$ mm, ${h}_{v}=-\,15$ mm, $\eta ={0}^{\circ }$. Then, the spectra of GDR at $\eta ={0}^{\circ }$ for both horizontal polarization (HP) and vertical polarization (VP), were acquired. As expected, the yield of GDR at $\eta ={0}^{\circ }$ is smaller than that of SPR, since only part of the beam’s Coulomb field interacts with the grating. The integration over a given spectral range gives the following radiated power in arbitrary units: SPR-HP $=\,31.04$, GDR-HP $=\,13.94$ and GDR-VP $\mathrm{=3.94}$. The GDR emitted by the semi-plane grating has approximately $2.2$ times less intensity than the SPR at $\eta {\mathrm{=\; 0}}^{\circ }$. Nevertheless, the observed spectral properties confirm that GDR is also monochromatic and its line widths $\le \mathrm{3 \% }$ for small angles $\eta$ and given $\varDelta \theta$ practically coincide with the same characteristics of SPR. For different grating orientation angles $\eta$, the GDR integral intensity of a few diffraction orders varies and reaches levels comparable to these of SPR as can be seen from the $\eta$-scans acquired in the range from $-{5}^{\circ }$ to ${20}^{\circ }$ while keeping ${h}_{v}\,\mathrm{=\; 0.5}$ mm, Fig. 2e.

**Figure 3:**
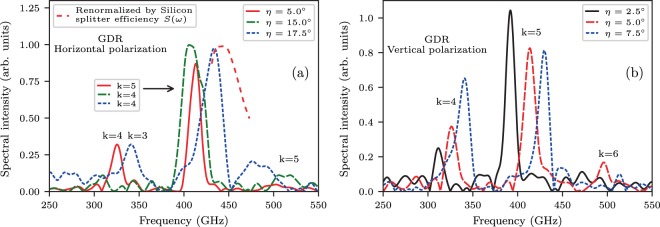
Typical GDR horizontal polarization (**a**) and vertical polarization (**b**) spectra as a function of the grating orientation angle $\eta$.

**Figure 4:**
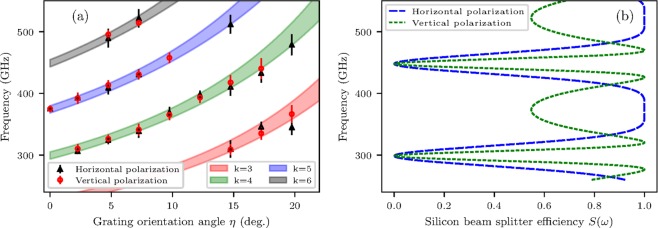
GDR spectral–angular distribution of $k=3\mbox{--}6$ diffraction orders calculated by Eqs. 1, 2 (**a** - colored bows) and measured GDR spectral peaks for both horizontal and vertical polarizations. Error-bars represent FWHM spectral line widths (**a** - markers). (**b**) Calculated efficiency of the $300$ $\mu$m-thick silicon beam splitter, angle of incidence ${45}^{\circ }$.

Investigation of the GDR properties on the grating orientation angle $\eta$ shows wide–range spectral tunability. Different polarizations of the GDR have similar spectral content with minor variation in intensity (Fig. 3, $\eta {\mathrm{=\; 5}}^{\circ }$ for both polarizations) due to the ${2}^{\circ }$–wedged sapphire vacuum window and different splitting efficiency $S(\omega )$ of the $300$ $\mu$m-thick silicon beam splitter used in the interferometer. The simulated dependence of $S(\omega )\propto 4\ast R\ast T$, where $R$ and $T$ are the reflection and transmission coefficients for two linear polarizations^28,29^, is shown in Fig. 4,b. In the case of ideal $\mathrm{50 \% }$ splitting $S(\omega )=1$.

An important GDR spectral feature is that not only central frequencies can be fine–tuned by selecting the $\eta$ angle, but also the spectral bandwidth for the same central frequency can be broadened by switching to lower diffraction orders at larger $\eta$. Typical frequency shifts for sequentially small $\eta$ angles are shown for GDR VP in Fig. 3b and the bandwidth increase near similar central frequencies are shown for GDR HP in Fig. 3a. In the current example, the bandwidth $\varDelta \lambda /\lambda$ was switched from $\mathrm{2.8 \% }$ to $\mathrm{10 \% }$ levels. According to splitter efficiency simulation Fig. 4b, $S(\omega )$ drops to zero at $300$ GHz and $450$ GHz. Around these frequencies Michelson interferometer gradually loses performance, but still produces auto–correlation with reduced visibility which can be recalculated to a spectrum via Fourier transform. However, spectral amplitude in this case is decreasing and can not be directly compared with amplitudes from other regions of $S(\omega )$ dependence. The red dashed line in Fig. 3a represents the re–normalized GDR HP spectra for $\eta {=17.5}^{\circ }$ which is valid to within an amplitude factor. The renormalization means addition of inversed spectral function multiplied by inversed splitter efficiency function, as can be derived from a generalized formula of Fourier transform spectroscopy^30,31^. This is done only for the GDR spectrum taken at $\eta {=17.5}^{\circ }$ in the range 430–470 GHz as other spectral lines lie in non-zero regions of $S(\omega )$ dependence.

A summary of the GDR spectra measurements for both HP and VP is shown in Fig. 4a along with the GDR spectral–angular distribution for diffraction orders $k=3\mbox{--}6$ calculated using Eq. 1. The full width at half maximum (FWHM) spectral widths are calculated by Eq. 2. Measured data are in close agreement with the dispersion relation Eq. 1.

## Discussion

It is expected that for the THz frequency range ($\lambda \sim 0.3$ mm) electrons with energy $10$ MeV can generate monochromatic GDR from a grating with $d\le 0.5$ mm and ${N}_{0}\ge 50$. In this case the spectral line may be tuned over a broad interval, thereby changing the grating orientation angle in the range $0 < \,\eta { < 20}^{\circ }$ with an accuracy of $\Delta \eta \sim {0.1}^{\circ }$ can result in a spectral range of 0.1–1 THz and a spectral accuracy ranging from sub-GHz to a few GHz depending on $k$ and $\eta$. Additional spectral selection can be performed by usage of bandpass filters^32^ or different grating profiles^33,34^.

The GDR radiated energy can be estimated through comparison of the GDR and CDR experimental data while CDR yield can be analytically found. Using the notation from ref. ^35^ one can write the following expression for CDR HP spectral density assuming a perfectly conducting semi-plane target and neglecting the terms lower than ${\gamma }^{-2}$ as:

3 $$\frac{d{W}_{CDR}^{HP}}{\hslash \,d\omega }={N}_{e}^{2}{\int }_{\varDelta {\theta }_{x},\varDelta {\theta }_{y}}\frac{{d}^{2}{W}_{0}^{HP}}{\hslash d\omega d\varOmega }F(\omega ,{\theta }_{x},{\theta }_{y})d\varOmega ;\,\frac{{d}^{2}{W}_{0}^{HP}}{\hslash d\omega d\varOmega }=\frac{\alpha }{4{\pi }^{2}}exp(-\frac{\omega }{{\omega }_{c}}\sqrt{1+{\gamma }^{2}{\theta }_{x}^{2}})\frac{{\theta }_{x}^{2}}{({\gamma }^{-2}+{\theta }_{x}^{2})({\gamma }^{-2}+{\theta }_{x}^{2}+{\theta }_{y}^{2})}$$

**Figure 5:**
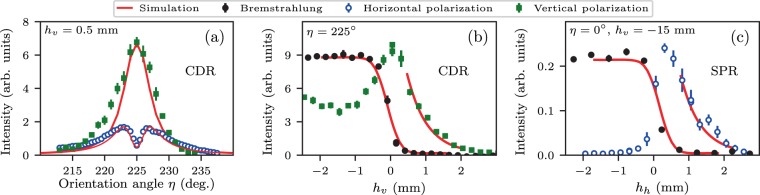
(**a**) Measured and simulated CDR angular distributions. (**b**) Measured and simulated bremsstrahlung and CDR VP intensity as a function of the vertical impact parameter ${h}_{v}$. (**c**) Measured and simulated coherent SPR HP intensity versus horizontal impact parameter ${h}_{h}$.

Here $F(\omega ,{\theta }_{x},{\theta }_{y})=exp(-({\omega }^{2}/{c}^{2})({\sigma }_{x}^{2}{\theta }_{x}^{2}+{\sigma }_{y}^{2}{\theta }_{y}^{2}+{\sigma }_{z}^{2}))$ is the 3D Gaussian beam form–factor, ${\omega }_{c}=\gamma c\mathrm{/2}{h}_{v}$ is the characteristic diffraction radiation frequency, $\omega =2\pi c/\lambda$ - radiation frequency, ${h}_{v}$ - vertical impact parameter, $\gamma$ - Lorentz factor, $\alpha$ - fine-structure constant, $c$ - speed of light, ${\theta }_{x}$ and ${\theta }_{y}$ are the projection angles between the specular reflection direction and the wave vector of the outgoing photon. Simulation of the CDR $\eta$-scans by Eq. 3 is presented in Fig. 5a - red curves which shows a reasonable agreement with the experimental data for the real electron bunch sizes and the detector angular acceptance neglecting electron beam divergence. To find the CDR HP emitted energy per bunch Eq. 3 should be integrated over the detected radiation frequency band as:

4 $$\varDelta {W}_{CDR}^{HP}={N}_{e}^{2}{\int }_{{{\rm{\nu }}}_{min}}^{{{\rm{\nu }}}_{max}}\frac{d{W}_{CDR}^{HP}}{\hslash \,d\omega }2\pi \hslash d{\rm{\nu }}\mathrm{}.$$

Assuming an angular acceptance of the detection system $\varDelta {\theta }_{x}\times \varDelta {\theta }_{y}\,=\,4\times 0.02\times 0.02$, bunch population ${N}_{e}=\,1.56\cdot {10}^{8}$, measured SBD detector sensitivity bandwidth $\varDelta {\nu }_{exp}=\,140\pm 1$ GHz (${\nu }_{min}=\,320$ GHz, ${\nu }_{max}=\,460$ GHz), electron bunch rms sizes in transverse and longitudinal directions ${\sigma }_{x}={\sigma }_{y}=\,300\pm 2$  µm and ${\sigma }_{z}=\,150\pm 5$ µm we obtain $\varDelta {W}_{CDR}^{HP}=\,120\pm 5$ pJ and, accordingly $\varDelta {W}_{CDR}^{HP}/\varDelta {\nu }_{exp}=\,860\pm 35$ pJ/THz. The results of the GDR HP measurements (Fig. 2e) are presented in the same arbitrary units as the CDR yield (Fig. 5a). For $\eta {=5}^{\circ }$ the measured intensity of the GDR HP $=\,0.24\pm 0.007$ is about $\mathrm{14.2 \% }$ of the CDR HP $=\,1.68\pm 0.2$ taken at $\eta {=223}^{\circ }$. For both cases ${h}_{v}\,=\,0.5$ mm. The spectral intensities ratio of GDR HP $k=5$ ($410$ GHz) to $k=4$ ($320$ GHz) in this case is $\mathrm{2.7:1}$ or $\mathrm{73 \% }$: $\mathrm{27 \% }$, (Fig. 3a). Hence, the intensity of the single GDR spectral line ${\nu }_{GDR}^{k\,=\,5}\,=\,410$ GHz may be estimated as $\mathrm{10.4 \% }$ of the $\varDelta {W}_{CDR}^{HP}$ and taking into account the GDR line width ($\varDelta {\nu }_{GDR}^{k\,=\,5}\,=\,15\pm 1$ GHz) one can obtain the estimation of the GDR spectral density as $\varDelta {W}_{GDR}^{HP}/\Delta {\nu }_{GDR}^{k\,=\,5}\,=\,832\pm 27$ pJ/THz. This means, that the intensity of a single coherent GDR line with $\varDelta \nu \le 15$ GHz from a train of $50$ short electron bunches with a total charge $Q \sim 50\times 25$ pC $\sim 1.25$ nC can achieve the level of $41.6$ nJ/THz. Such a source of monochromatic THz radiation based on a compact electron accelerator can be considered as a promising candidate for many practical applications. The errors include statistical errors due to the fitting or rms calculation in ${\sigma }_{x}$, ${\sigma }_{y}$, ${\nu }_{exp}$ and systematic errors from the uncertainty in ${\sigma }_{z}$.

Wide–range tunability and the possibility of bandwidth selection, along with further grating and electron beam parameter optimization will lead to a much higher peak radiation power. Due to the unperturbed interaction of the electron beam with the grating, one can consider GDR applications in time-resolved THz spectroscopy experiments^36^, to non–invasive beam diagnostics^37–39^ and multi–colour radiation generation^40^ by an array of gratings. To increase the GDR radiated power further, a slit–grating (i.e. when the electron beam is passing through a horizontal slit made in a grating) with an optimized profile should be considered.

## Methods

The experiment was performed at the KEK LUCX facility^41^. The detailed description of the accelerator, grating, and THz interferometer can be found in ref. ^25^. As it was discussed in ref. ^42^, the Michelson interferometer is optimized for a large bandwidth of both HP (polarization in the diffraction plane) and VP of the incoming radiation excluding only $300$ GHz and $450$ GHz spectral lines where silicon splitter efficiency $S(\omega )$ drops to zero. The SBD detector and a $100$ mm diameter wire-grid polarizer consisted of tungsten wires with diameters of $15$µm and spacings of $200$µm (installed in front of the SBD) were mounted to the rotation stage to allow for polarization selection during spectral measurements. All spectra (Figs. 2d and 3) were obtained by Fourier transform^43,44^ of the charge–normalized auto–correlation curves which were measured with an rms resolution $\varDelta \nu =c\mathrm{/2}L$ determined by the interferometer’s movable arm travel range $L\mathrm{=62}$ mm (zero–path difference point $\pm 31$ mm) as $\varDelta \nu /\nu \,\mathrm{=\;  < \; 0.8 \% }$ and so are plotted with identical scales. The Michelson interferometer used in the experiment has a resolution higher than that of the natural GDR VP spectral line width determined for a point-like aperture $\delta \nu /\nu \sim \mathrm{\ 0.89/}k{N}_{eff}\sim \mathrm{1.1 \% }$, since ${N}_{eff}\sim {N}_{0}\,\mathrm{=\; 15}$, $k\,\mathrm{=\; 5}$. However, the angular acceptance of the detection system $\varDelta \theta {\mathrm{=\; 1.6}}^{\circ }$ limited the measured spectral line widths to $\sim \mathrm{3 \% }$ level. The observed anomaly of the $\eta {\mathrm{=\; 15}}^{\circ }$ curve around $425$ GHz in Fig. 3a suggests to perform a higher resolution study to check for a more complex spectral structure.

Initially, the interferometer was set to a zero-path difference point and a number of preliminary scans were performed in order to verify the grating angle and the position with respect to the electron beam. The CDR yield versus the angle between the electron beam and the flat side on the rear of the grating ($\eta$-scan for a large angles around $\eta {\mathrm{=\; 225}}^{\circ }$) was measured for impact parameter ${h}_{v}\,\mathrm{=\; 0.5}$ mm, Fig. 5a. This allows the grating orientation angles $\eta$ to be determined with an accuracy better than ${1}^{\circ }$.

It was noted that the current experimental geometry for the investigation of the GDR properties has two impact parameters: vertical ${h}_{v}$ - the distance between the electron beam and the edge of the grating; and horizontal ${h}_{h}$ which can be defined as the distance between the electron beam and the grating main plane at $\eta {\mathrm{=\; 0}}^{\circ }$, Fig. 1. For $\eta$ corresponding to the maximum of CDR VP, (specular reflection direction from the flat rear side of the grating) the radiation yield dependence on the vertical impact parameter ${h}_{v}$ was obtained (Fig. 5b - green markers). The peak of the curve coincides with the target edge, the right slope corresponds to the CDR, and the left part is connected with CTR. A similar dependence for non-coherent radiation was measured in the optical wavelength range at the KEK ATF accelerator, and was discussed in ref. ^45^. In order to avoid direct beam interaction with the grating and to maintain a significant CDR/GDR yield, the vertical impact-parameter for further measurements was chosen as ${h}_{v}\,\mathrm{=\; 0.5}$ mm.

Electron beam parameter simulations^46,47^ show that the transverse rms bunch size at the grating location was equal to $300\pm 2$ µm. The measurements of the bremsstrahlung produced by electrons versus the vertical impact parameter ${h}_{v}$ confirm the simulation results (Fig. 5b - black markers). If the assumption is made that the transverse profile of the beam can be described by a Gaussian function (confirmed by measurements with luminescent screen installed $400$ mm downstream of the experimental station) the measured dependence can be described by the function:

5 $$F(y)\sim {\int }_{y}^{\infty }exp[-\,{({y}_{1}-{y}_{0})}^{2}\mathrm{/2}{\sigma }^{2}]d{y}_{1},$$

with free parameters ${y}_{0}\,\mathrm{=\; 33.15}\pm 0.1$ mm and $\sigma \,\mathrm{=\; 290}\pm 12$ µm.

The horizontal impact parameter ${h}_{h}\mathrm{=0.5}$ mm was set in accordance with the radiation yield dependence on the horizontal distance between the electron beam and the grating in SPR geometry: $\eta {\mathrm{=\; 0}}^{\circ }$, ${h}_{v}=-\,15$ mm, Fig. 5c. If ${h}_{h}$ is known, this allows for a comparison of yields at $\eta {\mathrm{=\; 0}}^{\circ }$ for GDR and SPR to be made.

## Data Availability

All data are available on request from the authors.
